# Antibiotic Consumption and Gram-Negative Resistance Dynamics in the ICU: A Five-Year Autoregressive Integrated Moving Average (ARIMA) Analysis

**DOI:** 10.7759/cureus.102615

**Published:** 2026-01-30

**Authors:** Lejla Rakovac Tupkovic, Nijaz Tihic, Jasmina Smajic, Hedim Osmanovic, Mirela Basic Denjagic, Predrag Jovanovic, Emir Becirovic, Minela Becirovic

**Affiliations:** 1 Department of Pharmacology, University Clinical Centre Tuzla, Tuzla, BIH; 2 Department of Microbiology, Faculty of Medicine, University of Tuzla, Tuzla, BIH; 3 Department of Microbiology, University Clinical Centre Tuzla, Tuzla, BIH; 4 Department of Anaesthesiology, Faculty of Medicine, University of Tuzla, Tuzla, BIH; 5 Department of Anesthesiology and Reanimation, University Clinical Centre Tuzla, Tuzla, BIH; 6 Department of Biostatistics, Faculty of Natural Sciences and Mathematics, University of Tuzla, Tuzla, BIH; 7 Department of Internal Medicine, Faculty of Medicine, University of Tuzla, Tuzla, BIH; 8 Department of Gastroenterology and Hepatology, University Clinical Centre Tuzla, Tuzla, BIH; 9 Intensive Care Unit, Internal Medicine Clinic, University Clinical Centre Tuzla, Tuzla, BIH; 10 Department of Physiology, Faculty of Medicine, University of Tuzla, Tuzla, BIH; 11 Department of Nephrology, Internal Medicine Clinic, University Clinical Centre Tuzla, Tuzla, BIH

**Keywords:** antimicrobial agents, antimicrobial drug resistance, arima model, atc/ddd methodology, gram negative bacteria

## Abstract

Aim: The aim of this study was to evaluate the impact of hospital antibiotic consumption on the rate of antimicrobial resistance (AMR) of gram-negative bacteria, specifically the *Enterobacteriaceae* family and the genus *Acinetobacter*, in the University Clinical Center (UCC) Tuzla, Bosnia and Herzegovina.

Methods: A five-year retrospective, observational, pharmacoepidemiological study was conducted (2014 to 2018). Antibiotic consumption was calculated using the WHO Anatomical Therapeutic Chemical/defined daily dose (ATC/DDD) methodology and expressed as DDD per 100 bed-days (BD). Microbiological data were obtained for *Klebsiella pneumoniae, Escherichia coli, Proteus mirabilis,* and *Acinetobacter *species. The temporal associations between consumption and resistance were analyzed using linear regression and autoregressive integrated moving average (ARIMA) models.

Results: The total antibiotic consumption at UCC Tuzla significantly increased from 61.35 to 73.51 DDD/100 BD (p=0.003). Consumption in intensive care units (ICUs) was significantly higher than the hospital-wide average (p<0.001), reaching up to 178.53 DDD/100 BD. ARIMA modeling confirmed significant positive correlations between the use of fluoroquinolones (J01MA) and resistance in *Acinetobacter baumannii *(beta = 7.678, p=0.006) and *K. pneumoniae* (beta = 18.368, p<0.001)9. A similar correlation was found for carbapenems (J01DD) and *E. coli* resistance (beta = 14.066, p=0.004).

Conclusion: The study demonstrates a significant temporal association between the volume of broad-spectrum antibiotic consumption and the escalation of AMR. The high selective pressure in ICUs identifies these units as primary reservoirs for multidrug-resistant pathogens. These findings highlight the importance of multidisciplinary antimicrobial stewardship programs and restricted use of reserve antibiotics to preserve therapeutic efficacy.

## Introduction

Antimicrobial resistance (AMR) is an evolutionary survival mechanism of bacteria, as old as the known world itself, dating back millions or even billions of years [1]. The selective pressure exerted by antibiotic use is a major contributor to AMR dynamics, particularly in gram-negative pathogens, which are responsible for a high proportion of hospital-acquired infections [2]. Gram-negative pathogens, such as *Escherichia coli, Klebsiella pneumoniae, Pseudomonas aeruginosa, and Acinetobacter baumannii*, are of particular concern as they increasingly develop resistance to nearly all available therapeutic options.

Hospital antibiotic consumption has been a critical area of research due to its implications for public health, antimicrobial resistance (AMR), and patient outcomes. Over the past decade, significant efforts have been made to monitor and regulate antibiotic use in healthcare settings across Europe [3]. Antibiotics are among the most commonly prescribed medications in hospitals, playing a vital role in treating bacterial infections. However, their overuse and misuse have contributed to the rise of AMR, which is a major public health concern.

Hospitals worldwide have been at the forefront of efforts to monitor and optimize antibiotic use, with initiatives such as the European Surveillance of Antimicrobial Consumption Network (ESAC-Net) [3] and the World Health Organization's surveillance systems [4], providing valuable data on consumption patterns. According to recent reports, antibiotic consumption in European hospitals varies significantly across countries. Northern European countries, such as Sweden and the Netherlands, tend to have lower rates of antibiotic use compared to Southern and Eastern European countries [5]. This variation is influenced by differences in healthcare systems, prescribing practices, and the prevalence of infectious diseases.

Intensive care units (ICUs) serve as epicenters for the development and dissemination of AMR [6]. Patients in these wards are subjected to high selection pressure due to the frequent use of broad-spectrum empirical antibiotic therapy. Excessive use of antimicrobial agents in these settings is directly linked to the selection of resistant clones, leading to increased mortality, prolonged hospital stays, and enormous economic costs.

While the correlation between antibiotic consumption and resistance is generally accepted, the dynamics of this relationship vary depending on local microbiological ecology. In Bosnia and Herzegovina, systematic data on monitoring this correlation using the Anatomical Therapeutic Chemical/defined daily dose (ATC/DDD) methodology [7] are still lacking, especially in tertiary care institutions. Therefore, the aim of this study was to analyze antibiotic consumption trends and examine their direct statistical association with the prevalence of resistant *Enterobacteriaceae* and* Acinetobacter* isolates over a five-year period (2014-2018).

The primary objective of this study was to analyze the temporal relationship between antibiotic consumption and resistance dynamics in gram-negative pathogens within the ICU using autoregressive integrated moving average (ARIMA) time-series modeling, to support evidence-based antimicrobial stewardship programs (ASPs). Secondary objectives included comparing consumption trends of high-priority antibiotics in the ICU versus hospital-wide data and evaluating the impact of specific stewardship-targeted classes (e.g., carbapenems, polymyxins, fluoroquinolones) on the emergence of multidrug-resistant (MDR) gram-negative strains.

## Materials and methods

Study design and population

This five-year retrospective, observational study was conducted at the University Clinical Center (UCC) Tuzla, a 1,200-bed tertiary care teaching hospital in Bosnia and Herzegovina, covering the period from January 2014 to December 2018.

The study included all patients admitted to the ICUs during the study period. The ICU was selected as the primary focus because it represents the epicenter of high-volume antibiotic use and the emergence of MDR gram-negative pathogens within the hospital. The inclusion criteria were all patients admitted to the UCC Tuzla for more than 24 hours during the five-year study period. The study exclusively focused on gram-negative pathogens (*Enterobacteriaceae* and *Acinetobacter*), as these represented the vast majority of clinical isolates in our ICUs.

The exclusion criteria were patients with a UCC Tuzla stay of less than 24 hours. Patients with gram-positive multidrug-resistant organisms (such as methicillin-resistant *Staphylococcus aureus* (MRSA) or vancomycin-resistant *Enterococci *(VRE)) were not included in the analysis due to their exceptionally low prevalence during the 2014-2018 period, ensuring the study’s focus remained on the most significant local epidemiological threats. The following were also excluded: (i) Screening swabs and surveillance cultures (to avoid overestimating resistance rates), (ii) duplicate isolates from the same patient within a 30-day period (only the first isolate was included).

Exposure and comparison

The primary exposure of interest was antibiotic consumption, analyzed specifically within the ICU and compared against hospital-wide consumption data to identify setting-specific trends. Data were obtained from the hospital’s central pharmacy database using the WHO ATC/DDD methodology, expressed as DDD per 100 bed-days (BD). Specific focus was placed on broad-spectrum antibiotics, including carbapenems, fluoroquinolones, third-generation cephalosporins, and polymyxins.

While antibiotic consumption data from non-ICU wards were included in the calculation of the hospital-wide average (total UCC consumption), these wards were not subjected to separate individual time-series modeling. The rationale for this was their low and relatively stable rates of antibiotic consumption, which lacked the necessary temporal variability required for robust ARIMA modeling compared to the high-pressure environment of the ICU wards.

Identification and testing

Identification and antimicrobial susceptibility testing were performed using the Vitek 2 automated system (bioMérieux SA, Marcy-l'Étoile, France) and interpreted according to the Clinical and Laboratory Standards Institute (CLSI) [8] or European Committee on Antimicrobial Susceptibility Testing 2018 (EUCAST) guidelines [9], valid during the study period.

Data analysis

The primary outcome was the prevalence of resistance, calculated as the percentage of non-susceptible clinical isolates (including both intermediate and resistant categories) relative to the total number of isolates tested for each specific pathogen, specifically focusing on the *Enterobacteriaceae* family (primarily *K. pneumoniae* and *E. coli*) and genus *Acinetobacter* (specifically *A. baumannii*). This continuous variable was used as the dependent variable in the time-series analysis to track the dynamics of resistance over the study period. MDR was defined as non-susceptibility to at least one agent in three or more antimicrobial categories. To manage the transition between CLSI and EUCAST guidelines during the 2014-2018 period, all susceptibility data were retrospectively re-evaluated using the 2018 EUCAST breakpoints to ensure internal consistency and eliminate artificial trends caused by shifting interpretive criteria.

Statistical analysis

Statistical analysis was performed using IBM SPSS Statistics for Windows, version 20.0 (IBM Corp., Armonk, New York, United States). To evaluate long-term trends and correlations, data were aggregated quarterly over the five-year study period. An ARIMA model was employed to account for temporal dependencies and filter out potential seasonal variations in antibiotic prescribing. The time-series stationarity was assessed using the Augmented Dickey-Fuller (ADF) test; first-order differencing (d=1) was applied where necessary to stabilize the mean. Model selection followed the Box-Jenkins approach, utilizing Autocorrelation Function (ACF) and Partial Autocorrelation Function (PACF) plots, with model parsimony prioritized via the Akaike Information Criterion (AIC). Missing data (less than 5%) were handled using linear interpolation. To evaluate the delayed impact of antibiotic consumption on resistance, a maximum lag of one quarter (three months) was pre-specified. A p-value of < 0.05 was considered statistically significant.

## Results

During the five-year study period, the analysis encompassed a total of 1,499,785 patient-days across the UCC Tuzla (which is the measure needed for DDD/ATC antibiotic consumption), of which 111,971 were recorded in ICUs. A total of 7,776 isolates of gram-negative bacteria were analyzed for antimicrobial susceptibility, of which 2662 were recorded in ICUs.

Antibiotic consumption trends

Overall antibiotic consumption at UCC Tuzla showed a statistically significant upward trend, increasing from 61.35 DDD/100 BD in 2014 to 73.51 DDD/100 BD in 2018 (p = 0.003). The consumption in ICUs was significantly higher than the hospital-wide average, with rates ranging between 130 and 180 DDD/100 BD (p < 0.001). Notable increases were observed in the use of reserve antibiotics, including fluoroquinolones (p = 0.001), polymyxins (p = 0.020), and glycopeptides (p = 0.004) (Table 1).

**Table 1:** Trends in antibiotic consumption at UCC Tuzla (2014–2018) Statistically significant: p < 0.05 DDD: Defined Daily Doses; BD: bed-days; ICU: intensive care unit; UCC: University Clinical Centre; ATC: Anatomical Therapeutic Chemical

ATC Group	2014 (DDD/100 BD)	2015 (DDD/100 BD)	2016 (DDD/100 BD)	2017 (DDD/100 BD)	2018 (DDD/100 BD)	Slope (β)	p-value	Trend
Total UCC Tuzla (J01)	61.35	63.08	66.74	68.54	73.51	2.98	0.003*	Increase
Fluoroquinolones (J01MA)	6.41	7.32	7.68	9.65	10.61	1.073	0.004*	Increase
Glycopeptides (J01XA)	0.75	0.96	1.15	1.32	1.69	0.224	0.001*	Increase
Polymyxins (J01XB)	0.3	0.15	0.54	0.98	1.37	0.297	0.020*	Increase
Carbapenems (J01DH)	1.5	1.78	2.08	2.27	2.42	0.232	0.001*	Increase
ICU Consumption Range	139	132.8	127.7	162.1	178.5	10.83	0.108	Stable/High

MDR pathogens

MDR prevalence remained critically high throughout the study period, particularly in the ICU settings. *A. baumannii* maintained near-universal resistance levels, exceeding 90% in most ICU units. *K. pneumoniae* showed a significant rising trend in MDR frequency, peaking at over 50% in 2017. *E. coli* was the only pathogen with a statistically significant hospital-wide increase in MDR strains, rising from 5.48% to 12.44% (p = 0.002) (Table 2).

**Table 2:** Prevalence and trends of multidrug-resistant strains at UCC Tuzla (2014–2018) Statistically significant (p < 0.05) ICU: intensive care unit; UCC: University Clinical Centre

Pathogen	2014 (%)	2015 (%)	2016 (%)	2017 (%)	2018 (%)	Slope (β)	p-value	Trend
UCC Tuzla (Overall)								
Escherichia coli	5.48	6.1	8.79	10.25	12.44	1.807	0.002*	Increase
Klebsiella pneumoniae	38.43	41.3	38.99	51.35	48.42	3.003	0.094	Stable
Acinetobacter baumannii	87.97	88.72	88.32	86.77	92.68	0.747	0.361	Stable
ICU Settings								
Acinetobacter baumannii	100	85.71	86.67	100	90.91	-0.389	0.888	Stable
Klebsiella pneumoniae	0	0	25	54	60	17.4	0.009*	Increase

Correlation between consumption and resistance (ARIMA model)

ARIMA modeling confirmed significant positive correlations between the use of fluoroquinolones (J01MA) and resistance in *A. baumannii* (beta = 7.678, p=0.006) and *K. pneumoniae* (beta = 18.368, p<0.001)9. A similar correlation was found for carbapenems (J01DD) and *E. coli* resistance (beta = 14.066, p=0.004) (Table 3).

**Table 3:** Causal correlation between antibiotic consumption and MDR resistance (ARIMA model) Statistically significant: p < 0.05 ARIMA: autoregressive integrated moving average; MDR: multidrug-resistant

Dependent Variable (MDR Resistance)	Independent Variable (Antibiotic Class)	t-statistic	Slope (β)	p-value
*Acinetobacter baumannii*	Fluoroquinolones (J01MA)	3.094	7.678	0.006*
	Nitrofurans/Polymyxins (J01XA)	2.625	5.143	0.017*
*Klebsiella pneumoniae*	Fluoroquinolones (J01MA)	4.517	18.368	<0.001*
	Aminoglycosides (J01GB)	2.196	2.428	0.041*
	Glycopeptides (J01XA)	3.249	11.492	0.004*
*Escherichia coli*	Carbapenems (J01DD)	3.328	14.066	0.004*
	Total Antibiotic Use (J01)	2.894	0.149	0.009*
	Sulfonamides (J01EE)	4.539	2.26	<0.001*

## Discussion

Trends in antibiotic consumption

Overall Consumption Patterns

The findings from UKC Tuzla provide compelling empirical evidence that complements and, in certain aspects, contrasts with broader European trends reported by ESAC-Net [5]. While aggregated European data suggest relative stabilization of hospital antibiotic consumption over the past decade, the statistically significant increase observed at UKC Tuzla from 61.35 to 73.51 DDD/100 BD between 2014 and 2018 indicates a sustained upward trajectory within this tertiary-care setting. This divergence underscores the importance of institution-specific analyses, as continental averages may obscure clinically meaningful local dynamics.

The markedly higher antibiotic consumption recorded in ICUs, reaching 130-180 DDD/100 BD, aligns with established evidence that ICUs represent epicentres of antimicrobial exposure [10]. The magnitude of ICU-associated consumption at UKC Tuzla significantly exceeds hospital-wide averages and reflects a clinical environment characterized by severe illness, high diagnostic uncertainty, and frequent reliance on empiric broad-spectrum therapy. These conditions collectively amplify selective pressure, fostering the emergence and persistence of multidrug-resistant organisms [11]. Particularly concerning is the documented increase in the use of reserve antibiotics, including fluoroquinolones, polymyxins, and glycopeptides. The statistically significant upward trends in these agents suggest progressive therapeutic escalation, likely driven by rising resistance to first-line regimens. While such escalation may be clinically justified at the individual patient level, at the population level, it contributes to a self-reinforcing cycle of resistance selection and diminishing therapeutic options.

Data from ESAC-Net and other sources indicate that antibiotic consumption in European hospitals has remained relatively stable over the past decade, with some fluctuations. However, there are notable differences between countries and regions [4]. For example, countries with robust ASPs have seen a decline in antibiotic use, while others have experienced an increase [12]. Hospital antibiotic consumption has risen steadily worldwide [13], driven by factors such as the growing prevalence of infections, the complexity of medical treatments, and the widespread use of empiric therapy. Broad-spectrum antibiotics, including third- and fourth-generation cephalosporins, fluoroquinolones, and carbapenems, are frequently prescribed in hospitals due to their effectiveness against a wide range of pathogens. However, their overuse has been linked to the selection of resistant strains. For example, carbapenem use has been strongly associated with the emergence of carbapenem-resistant *Enterobacteriaceae* (CRE) [14], which are often resistant to multiple antibiotic classes.

The rise in antibiotic consumption has had profound implications for AMR in gram-negative bacteria [15]. These organisms are particularly concerning due to their ability to acquire and disseminate resistance genes through plasmids and other mobile genetic elements. For instance, the spread of extended-spectrum beta-lactamase (ESBL)-producing *E. coli *and *Klebsiella species* has been linked to the overuse of cephalosporins [16]. Similarly, the increasing use of carbapenems has driven the emergence of carbapenemase-producing organisms, which are often resistant to nearly all available antibiotics. Gram-negative bacteria are also known for their ability to develop resistance through mechanisms such as efflux pumps, enzymatic degradation, and target site modifications [17]. The overuse of antibiotics in hospitals creates selective pressure that favors the survival and proliferation of resistant strains. This is particularly problematic in ICUs, where patients are often critically ill and receive prolonged courses of antibiotics, creating an environment conducive to the spread of resistant pathogens.

Antibiotic Consumption and the Ecology of Gram-Negative Resistance

The resistance patterns observed at UKC Tuzla strongly support the ecological link between antibiotic consumption and AMR in gram-negative bacteria. In our study, *A. baumannii* demonstrated persistently extreme resistance levels, exceeding 90% in most ICU units, consistent with its well-documented capacity to survive under intense antimicrobial pressure and hospital environmental stressors. Similarly, the rising MDR prevalence in* K. pneumoniae*, peaking above 50% in 2017, mirrors regional and global trends associated with the dissemination of ESBL- and carbapenemase-producing clones [18]. Notably, *E. coli* was the only pathogen exhibiting a statistically significant hospital-wide increase in MDR prevalence, rising from 5.48% to 12.44%. This finding is particularly important, as it suggests that resistance selection is no longer confined to high-risk units such as ICUs but is extending into broader hospital ecosystems. Such expansion may reflect cumulative antibiotic exposure across wards and highlights the role of horizontal gene transfer in facilitating resistance dissemination beyond traditionally high-burden settings.

Quantitative Link Between Consumption and Resistance: ARIMA-Based Evidence

The study demonstrated significant positive correlations between fluoroquinolone use and resistance in both *A. baumannii* and *K. pneumoniae*, as well as between carbapenem consumption and *E. coli* resistance. These findings, supported by ARIMA time-series modeling, suggest strong ecological and temporal evidence consistent with a causal relationship, and reinforce the concept that sustained exposure to specific antibiotic classes acts as a powerful driver of resistance amplification [19]. Fluoroquinolones, in particular, are known to exert broad collateral selective effects, promoting resistance not only through target site mutations but also by inducing efflux pump overexpression and facilitating plasmid-mediated resistance [20]. The observed beta coefficients highlight the magnitude of this effect and emphasize the disproportionate ecological costassociated with these agents. 

Impact of the COVID-19 Pandemic

The COVID-19 pandemic had a significant impact on antibiotic consumption in European hospitals [21]. In the early stages of the pandemic, there was a surge in antibiotic use due to uncertainties about the management of COVID-19 patients and concerns about secondary bacterial infections. However, as evidence emerged that bacterial co-infections were less common than initially thought, antibiotic use declined in many hospitals. The pandemic also highlighted the importance of antimicrobial stewardship in crisis situations. Hospitals that had strong ASPs in place were better equipped to manage antibiotic use during the pandemic, avoiding unnecessary prescriptions and minimizing the risk of resistance.

Factors influencing antibiotic consumption

The consumption trends observed at UCC Tuzla reflect a complex interplay of institutional, prescriber-related, and patient-specific factors. The observed increase in reserve antibiotic consumption may be attributed, at least in part, to inadequate adherence to antimicrobial stewardship recommendations, despite the presence of a formal stewardship program. International experience demonstrates that stewardship maturity is a key determinant of consumption trajectories [22], and the UCC Tuzla data provide a strong empirical rationale for strengthening stewardship infrastructures.

Prescriber behavior, shaped by clinical risk aversion and limited access to rapid diagnostics, may further drive empiric escalation, particularly in critically ill populations. At the same time, the high burden of elderly patients with multiple comorbidities inevitably increases antibiotic exposure, underscoring the need for precision-based antimicrobial strategies rather than indiscriminate restriction.

The convergence of rising antibiotic consumption and increasing MDR prevalence at UCC Tuzla exemplifies the clinical and public health consequences of unchecked antimicrobial pressure. MDR infections are associated with prolonged hospitalization, increased mortality, and escalating healthcare costs, placing substantial strain on already resource-limited health systems. Importantly, the findings suggest that resistance containment cannot be achieved solely through reactive therapeutic escalation. Instead, proactive optimization of antibiotic use guided by stewardship principles, local epidemiology, and quantitative consumption-resistance modelling is essential to prevent therapeutic nihilism and preserve antimicrobial efficacy.

Antimicrobial Stewardship Programs

ASPs are a key factor in shaping antibiotic consumption trends in European hospitals [23]. These programs aim to optimize antibiotic use by promoting evidence-based prescribing, reducing unnecessary prescriptions, and minimizing the risk of resistance. Countries with well-established ASPs, such as the Netherlands and Sweden, have seen significant reductions in antibiotic consumption [12,24,25]. ASPs typically involve a multidisciplinary team of healthcare professionals, including infectious disease specialists, pharmacists, and microbiologists. They use a range of strategies, such as guidelines, audits, and feedback, to improve prescribing practices. The success of these programs depends on strong leadership, adequate resources, and the engagement of healthcare staff.

National Policies and Guidelines

National policies and guidelines play a crucial role in regulating antibiotic use in hospitals [26]**.** Many European countries have developed national action plans to combat antimicrobial resistance, which include measures to reduce antibiotic consumption. These plans often involve setting targets for antibiotic use, promoting the use of narrower-spectrum antibiotics, and encouraging the implementation of ASPs. The European Union has also taken steps to address AMR through initiatives such as the European One Health Action Plan against Antimicrobial Resistance [27]. This plan emphasizes the need for a coordinated approach to antibiotic use across human and animal health sectors.

Prescriber Behavior and Education

The prescribing behavior of healthcare professionals is a major determinant of antibiotic consumption in hospitals. Factors such as clinical experience, knowledge of antibiotic guidelines, and attitudes towards resistance can influence prescribing decisions [28]. Education and training are essential for promoting rational antibiotic use and ensuring that prescribers are aware of the latest evidence and guidelines. In recent years, there has been a growing emphasis on the role of education in antimicrobial stewardship. Many hospitals now provide regular training sessions for healthcare staff, covering topics such as antibiotic resistance, appropriate prescribing, and the principles of stewardship.

Patient Factors

Patient factors, such as age, comorbidities, and the severity of illness, can also influence antibiotic consumption in hospitals. Older patients and those with chronic conditions are more likely to receive antibiotics due to their increased risk of infections. However, these patients are also more vulnerable to the adverse effects of antibiotics, including resistance and side effects. Hospitals are increasingly adopting personalized approaches to antibiotic therapy, taking into account individual patient factors and the results of diagnostic tests [29]. This approach, known as precision medicine, aims to optimize treatment outcomes while minimizing the risk of resistance.

Implications of antibiotic consumption trends

Antimicrobial Resistance

The most significant implication of antibiotic consumption trends is their impact on AMR. High levels of antibiotic use in hospitals contribute to the selection and spread of resistant bacteria, making infections more difficult to treat [30]. This has serious consequences for patient outcomes, healthcare costs, and public health. European hospitals are facing increasing challenges from multidrug-resistant organisms (MDROs), such as MRSA and CRE [31]. These infections are associated with higher mortality rates, longer hospital stays, and increased healthcare costs. Reducing antibiotic consumption is essential for slowing the spread of resistance and preserving the effectiveness of existing antibiotics.

Healthcare Costs

Antibiotic consumption also has significant economic implications for healthcare systems. The cost of antibiotics themselves is relatively low, but the consequences of resistance can be substantial. Infections caused by resistant bacteria often require more expensive treatments, longer hospital stays, and additional diagnostic tests. By optimizing antibiotic use, hospitals can reduce healthcare costs and improve the efficiency of resource allocation. ASPs have been shown to be cost-effective, with savings outweighing the costs of implementation [32].

Patient Outcomes

Appropriate antibiotic use is essential for ensuring positive patient outcomes. Overuse and misuse of antibiotics can lead to adverse effects, such as *Clostridioides difficile* infections, which are associated with significant morbidity and mortality. On the other hand, underuse or delayed use of antibiotics can result in treatment failure and worse outcomes for patients with serious infections. ASPs aim to strike a balance between these extremes, ensuring that patients receive the right antibiotic, at the right dose, for the right duration. This approach has been shown to improve patient outcomes and reduce the risk of resistance. Beyond the emergence of antimicrobial resistance, the intensive use of certain antibiotics is associated with severe, potentially fatal systemic adverse reactions. A prominent example is the Drug Reaction with Eosinophilia and Systemic Symptoms (DRESS) syndrome, a type IV hypersensitivity reaction. While various antibiotics can trigger this condition, vancomycin has been increasingly identified as a primary cause. As highlighted in a recent systematic review by Bhumireddy et al. (2025), vancomycin-induced DRESS syndrome carries significant clinical risks, underscoring the necessity for vigilant monitoring and robust antibiotic stewardship in the ICU setting [33].

Future directions

The future of antibiotic consumption in European hospitals will depend on the continued strengthening of ASPs. This includes expanding the reach of these programs to all healthcare settings, improving data collection and monitoring, and promoting the use of rapid diagnostic tests to guide antibiotic therapy. Research and innovation are essential for addressing the challenges of antibiotic consumption and resistance. This includes the development of new antibiotics, alternative therapies, and diagnostic tools. European hospitals are increasingly participating in clinical trials and research initiatives to advance these efforts. Collaboration between healthcare professionals, policymakers, and researchers is crucial for tackling the complex issue of antibiotic consumption. European hospitals can benefit from sharing best practices, data, and resources through networks such as ESAC-Net and the European Centre for Disease Prevention and Control (ECDC) [34].

Limitations

Despite its longitudinal depth, this study has several limitations. First, as an ecological study using aggregated data, it is subject to the ecological fallacy; correlations observed at the population level (ICU-wide) do not necessarily reflect individual patient-level experiences. Since patient-level data (e.g., individual dose, duration, and indication) were unavailable, we could not account for specific clinical confounders. Second, while the ARIMA model accounts for temporal dependencies, it cannot fully control for non-prescribing factors such as shifts in infection control protocols, changes in diagnostic thresholds, or localized outbreaks that may have influenced resistance dynamics independently of antibiotic consumption. Finally, this study is retrospective in design and confined to a single tertiary-care center (UCC Tuzla) over the 2014-2018 period, which inherently limits the generalizability of the findings to outpatient settings or other regional healthcare contexts. Potential sources of bias include truncated datasets and a primary focus on MDR organisms without a more granular stratification into extensively drug-resistant (XDR) or pandrug-resistant (PDR) categories. Finally, the lack of post-2018 data restricts insight into more recent resistance trends, particularly in the context of altered prescribing practices during and after the COVID-19 pandemic, which will be analysed in the near future.

## Conclusions

This five-year study highlights the intense antimicrobial pressure and concerning resistance dynamics within the ICU of a large tertiary care hospital, mirroring the broader complexities of hospital antibiotic consumption across Europe. While regional variability and healthcare infrastructure challenges persist, our findings provide a strong ecological and temporal relationship between the consumption of broad-spectrum antibiotics, specifically carbapenems and fluoroquinolones, and the emergence of MDR gram-negative pathogens. Although the ecological nature of this analysis precludes the establishment of a direct causal link at the individual patient level, the use of ARIMA time-series modeling underscores how excessive selective pressure drives the prevalence of resistant isolates in critical care settings. Understanding consumption patterns and the ecological drivers of resistance is essential for designing targeted interventions that mitigate resistance while safeguarding patient outcomes.
